# Analyzing Cattle Activity Patterns with Ear Tag Accelerometer Data

**DOI:** 10.3390/ani14020301

**Published:** 2024-01-18

**Authors:** Shuwen Hu, Antonio Reverter, Reza Arablouei, Greg Bishop-Hurley, Jody McNally, Flavio Alvarenga, Aaron Ingham

**Affiliations:** 1 Agriculture and Food, CSIRO, Saint Lucia, QLD 4067, Australia; toni.reverter-gomez@csiro.au (A.R.); greg.bishop-hurley@csiro.au (G.B.-H.); jody.mcnally@csiro.au (J.M.); aaron.ingham@csiro.au (A.I.); 2 Data61, CSIRO, Pullenvale, QLD 4069, Australia; reza.arablouei@csiro.au; 3 NSW Department of Primary Industries, Armidale, NSW 2350, Australia; flavio.alvarenga@dpi.nsw.gov.au

**Keywords:** accelerometer data, cattle diurnal activity, daily differential activity, animal welfare, wearable sensor

## Abstract

**Simple Summary:**

We used smart ear tags with accelerometers to characterise the 24 h activity profiles of Angus and Brahman steers in different environments. The activity metric was calculated from accelerometer data that were either unprocessed or subject to a high-pass filtering method to remove the effect of gravity. We show that the median provides a robust measure of activity profiles over each day and the benefits of filtering accelerometer data. Distinct activity patterns can be seen between the different environments, but further studies are required to understand the impacts of cattle breed, environment and management practices.

**Abstract:**

In this study, we equip two breeds of cattle located in tropical and temperate climates with smart ear tags containing triaxial accelerometers to measure their activity levels across different time periods. We produce activity profiles when measured by each of four statistical features, the mean, median, standard deviation, and median absolute deviation of the Euclidean norm of either unfiltered or high-pass-filtered accelerometer readings over five-minute windows. We then aggregate the values from the 5 min windows into hourly or daily (24 h) totals to produce activity profiles for animals kept in each of the test environments. To gain a better understanding of the variation between the peak and nadir activity levels within a 24 h period, we divide each day into multiple equal-length intervals, which can range from 2 to 96 intervals. We then calculate a statistical measure, called daily differential activity (DDA), by computing the differences in feature values for each interval pair. Our findings demonstrate that patterns within the activity profile are more clearly visualised from readings that have been subject to high-pass filtering and that the median of the acceleration vector norm is the most reliable feature for characterising activity and calculating the DDA measure. The underlying causes for these differences remain elusive and is likely attributable to environmental factors, cattle breeds, or management practices. Activity profiles produced from the standard deviation (a feature routinely applied to the quantification of activity level) showed less uniformity between animals and larger variation in values overall. Assessing activity using ear tag accelerometers holds promise for monitoring animal health and welfare. However, optimal results may only be attainable when true diurnal patterns are detected and accounted for.

## 1. Introduction

Wearable sensors such as triaxial accelerometers provide a reliable, energy-efficient, and affordable means for monitoring changes in animal activity level and behaviour and as such may play a role in the management of animals through precision livestock farming (PLF) [1,2]. Animal activity can be defined as the measure of all physical movement within a set time period [3], whereas behaviour is defined as a specific conduct or response of an animal (such as grazing or ruminating) [4]. PLF approaches provide the opportunity for continuous and objective monitoring of individual animals and may be used for the assessment of animal health and welfare, productivity, sustainability, and overall farm management [5,6,7,8]. Data collected by accelerometers have been used to classify livestock behaviours such as grazing, ruminating, and walking [9,10,11,12,13], as well as predict biting and chewing rates [14,15]. Accelerometer data can also be used to quantify activity levels, representing the cumulative forces measured over a specific time window. Such activity measures have been applied in detecting early signs of respiratory diseases in cattle by tracking changes in activity levels [2,16,17]. Accelerometers attached to ear tags have been utilised for monitoring physical activity and detecting oestrus in dairy cows [18,19,20,21]. Commercial ear tag accelerometers have also been used to identify behaviours and activity levels of grazing cattle [22,23]. The success of the associated data-driven solutions depends largely on the quantity and quality of the available ground-truth data used for model training as well as the features extracted from the accelerometer data [24,25].

Accelerometer readings can be transformed into measures of animal activity following a mathematical calculation in which the readings of three axes *x*, *y*, and *z*, representing different orthogonal directions, can be combined to calculate an acceleration magnitude that is independent of orientation [24,26]. Alternatively, activity can be calculated from different statistical features derived from the triaxial accelerometer readings, such as the mean, standard deviation (SD), median, and median absolute deviation (MAD). Mean and SD of acceleration are two widely used statistical features. The mean values of each axis are useful for discriminating the animal behaviour classes, especially for the *x* and *z* axes [27]. The SD of the acceleration magnitude is a measure of the variability of the acceleration within a specific window. It provides an indication of how consistent the intensity of movement is during any activity [28]. According to Lyons et al. [29], the SD of the acceleration is a valuable feature for distinguishing between static and dynamic activities/behaviours. The median is regarded as a more robust statistical feature compared to the mean as it is less sensitive to outliers and erroneous readings [13]. Support vector machine algorithms have selected the median of the acceleration magnitude as the optimal feature when classifying human behaviours such as walking, ascending stairs, and descending stairs using triaxial accelerometer data [30]. The median absolute deviation (MAD) is the median of the absolute difference between all data points and their median. The MAD is a measure of spread and can be less sensitive to outliers than SD [31].

Accelerometer data can be analysed without processing or following a filtering step. A high-pass filter is a preprocessing step for the accelerometer data analysis removing low frequencies, which aims to obtain the acceleration without the influence of gravity [32]. Riaboff et al. [33] evaluate the effects of filters including low-pass filters and high-pass filters on the prediction of cattle behaviour from accelerometer data. They highlight that the high-pass filter had the most significant effect on the classification accuracy and suggest signal filtering has the potential to dramatically reduce the performance of prediction. A high-pass filter removes frequencies lower than the chosen cut-off frequency, and there is no consistency in the choice of the cut-off frequency. Arablouei et al. [27] tune the filter coefficient as a hyperparameter and they suggest removing the frequencies lower than 2.78 HZ.

Animals can exhibit a distinct daily pattern of low and high activity. In the case of cattle, it is common to observe a higher activity during the early morning and late afternoon. Conversely, activity levels are lower during the night [34,35]. Daylight hours are primarily allocated to grazing behaviour, while rumination and resting behaviours are more common during nighttime [36]. Nevertheless, the diurnal rhythm (activity over 24 h) of individual animals can vary depending on management or environmental factors [3,37]. For example, accelerometer data have been used to analyse how cattle respond to environmental heat [38]. Before identifying any anomaly in accelerometer data, it is crucial to understand the animals’ diurnal activity rhythm [3,17,39]. Wagner et al. [40] detected changes in the diurnal activity rhythm of cattle related to diseases, stress, and reproductive events using the CowView system, which utilises nonwearable sensors. Shahriar et al. [41] utilised wearable accelerometer sensors attached to collars to identify increased activity levels associated with abnormal oestrus events. These studies highlight the significance of characterising activity patterns and their associations with various physiological events using sensor technology.

The primary objective of this study was to assess the effects of high-pass filtering on the calculations of cattle activity using four different statistical features calculated from triaxial accelerometer readings. A secondary objective of this study was to examine different measures of activity, calculated hourly, daily, or as the difference between the highest and lowest activity levels within a 24 h period and assess variations between individual animals within herds and across different environments.

## 2. Materials and Methods

### 2.1. Cattle Populations and Data Collection

Data were collected during two experiments: (1) at the CSIRO FD McMaster Laboratory Chiswick, 25 km south of Armidale, NSW, Australia (temperate climate, 30.6063^∘^ S, 151.5443^∘^ E) with 7 Angus steers over 9 days in March 2020, and (2) 50 km south of Townsville, QLD, Australia at the CSIRO Lansdown Research Station (tropical climate, 19.6573^∘^ S, 146.8349^∘^ E) with 11 Brahman steers over 30 days in August–September 2020. The average minimum temperature in March 2020 in Armidale was 4.5 ^°^C, while the average maximum temperature in August–September 2020 in Lansdown was 32.3^°^C. We provide the summary statistics of the temperature data for the periods when the experiments took place in Armidale and Lansdown in Supplementary Table S1. Approvals for animal care and handling protocols in these two experiments were received from the CSIRO FD McMaster Laboratory Chiswick Animal Ethics Committee with the animal research authority number 19/18 and the CSIRO Queensland Animal Ethics Committee with the project approval number 2019-34, separately.

**Figure 1:**
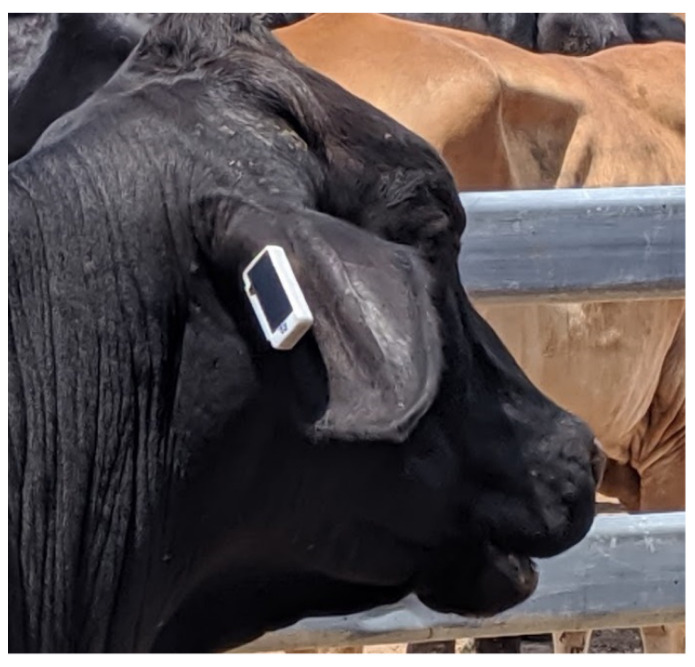
Angus cattle with ear tag (adapted from Wang et al. [12]).

In Armidale, cattle were yarded daily at approximately 16:00 and then released at 09:00 the following morning into a 1 ha paddock. The overnight yards were approximately 10 m by 10 m and contained a pasture base and water available in troughs. However, pasture was limited; hence, extended periods of grazing during this stay were unlikely. The daily grazing took place in 1 ha paddocks containing healthy perennial ryegrass, allowing for unrestricted grazing by the cattle. In Lansdown, the cattle had unrestricted access to mixed tropical pasture in a 15 ha paddock for the duration of the trial. In March, the sunrise and sunset times in Armidale are approximately 06:45 and 19:20. In August/September, these times in Lansdown are approximately 05:55 and 18:10. Cattle were fitted with an ear tag containing an accelerometer (Figure 1), collecting readings at 62.5 Hz. More details about this ear tag can be found in Wang et al. [12].

### 2.2. Activity Levels

Daily periods were defined as the 24 h spanning 00:00 h to 23:59 h. To ensure equal representation of all time periods, only accelerometer data that represented a continuous 24 h period were retained for analysis. This resulted in datasets of 7 days for the 7 Angus cattle in the Armidale trial and 20 days for the 11 Brahman cattle in the Lansdown trial.

At the set frequency (62.5 Hz in our case), the accelerometers generated data for three axes (*x*, *y* and *z*), which were denoted as $x_{t}$ , $y_{t}$ , and $z_{t}$ , respectively. The magnitude of the acceleration was defined as follows:

(1) $${ACC}_{t}=\sqrt{x_{t}^{2}+y_{t}^{2}+z_{t}^{2}}.$$

We calculated the mean, median, SD, and MAD values of ${acc}_{t}$ for five-minute windows. The SD of ${ACC}_{t}$ in a specific window is consistent with the movement intensity [28]. We considered these four statistical features as potential measures of activity and compare their performance.

As we demonstrated previously in detail [27], a first-order high-pass Butterworth filter can be applied to remove the effect of gravity from accelerometer readings efficiently. Applying this filter to the accelerometer readings in three axes is expressed as

$$\begin{array}{c} {\tilde{x}}_{t}=\gamma {\tilde{x}}_{t-1}+x_{t}-x_{t-1}\\ {\tilde{y}}_{t}=\gamma {\tilde{y}}_{t-1}+y_{t}-y_{t-1}\\ {\tilde{z}}_{t}=\gamma {\tilde{z}}_{t-1}+z_{t}-z_{t-1} \end{array}$$

where γ is the filter parameter set to 0.75 and ${\tilde{x}}_{t}$ , ${\tilde{y}}_{t}$ , and ${\tilde{z}}_{t}$ are the filter outputs at time index *t*. Subsequently, we calculated the filtered acceleration as

(2) $${\tilde{ACC}}_{t}=\sqrt{{\tilde{x}}_{t}^{2}+{\tilde{y}}_{t}^{2}+{\tilde{z}}_{t}^{2}}.$$

We denoted the median of ${\tilde{ACC}}_{t}$ over any 5 min window indexed by *i* as ${med}_{i}$ . We also calculated the MAD of the filtered acceleration as

$${MAD}_{i}=median(|{\tilde{ACC}}_{t}-{med}_{i}\left|\right)$$

Similarly, we computed the mean and SD of the filtered acceleration within five-minute windows to facilitate the comparison of features.

The hourly activity in total was calculated for each of the features through the sum of the respective five-minute window values. The mean hourly activity was then calculated for each hour over the duration of the trial (7 days in Armidale and 20 days in Lansdown) and the consecutive values for the 24 h period are shown to produce the daily activity profile.

### 2.3. Daily Differential Activity

**Figure 2:**
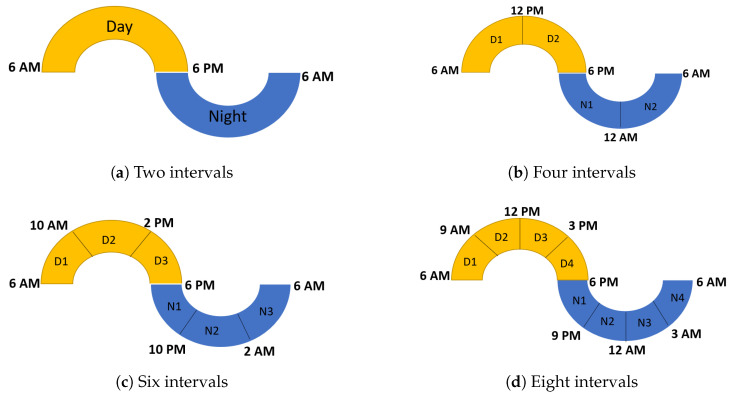
Schematic illustration of 2, 4, 6, and 8 intervals in a 24 h period.

To study the differences between the highest and the lowest daily activity intervals, we divided each 24 h into day and night periods according to sunrise and sunset times. As mentioned, the sunrise and sunset times were 06:45 and 19:20 in March in Armidale, and 05:55 and 18:10 in August/September in Lansdown. Hence, in both experiments, day and night each consisted of 12 h. However, to better recognise animal activity differences between the highest and lowest activity intervals, we further divided each 24 h period into intervals of equal length. These included 4 intervals of 6 h, 6 intervals of 4 h, 8 intervals of 3 h, 12 intervals of 2 h, 24 intervals of 1 h, 48 intervals of 30 min, and 96 intervals of 15 min. For illustration purposes, Figure 2 shows the schematic representation for partitions of 2, 4, 6, and 8 intervals.

For a given set of intervals, we calculated the DDA measure as the activity difference for all pairwise intervals (e.g., D1–N1 and N1–D1, with 12 possible pairs for the 4-interval case). Table 1 shows the total number of possible pairs for each set of intervals. We defined the three components of the DDA as follows: (1) DDA_p: the pair generating the maximum difference between the highest daily activity and the lowest daily activity, (2) DDA_v: the value of the maximum difference, and (3) DDA_e: the information entropy.

DDA_p and DDA_v were, respectively, alphanumeric and numeric variables indicating the animal behaviour in terms of the intervals where the extreme activity difference was observed in a 24 h day. DDA_e measured the regularity of the behaviour of extreme activity difference during the entire course of the experiment (i.e., 7 and 20 days in Armidale and Lansdown, respectively). Therefore, for each animal, a DDA_p value and a DDA_v value could be calculated for each interval on every day. All the DDA_v values for an animal could be averaged across all days and the frequency distribution of all DDA_p values for an animal could be calculated to facilitate the computation of DDA_e as follows

(3) $$DDA\_e=-\sum_{d=1}^{M}P\left(X_{d}\right){log}_{2}P\left(X_{d}\right)$$

where *M* is the number of pairs for an animal, and $P(X_{d})$ is the probability of a specific pair occurring within the total number of pairs. We also have $\sum_{d=1}^{M}P\left(X_{d}\right)=1$ .

Contextually, we can anticipate a strong negative correlation between DDA_v and DDA_e. At one extreme, a very regular animal (DDA_e = 0) will have the same DDA_p across all days. On the other extreme, an animal with little change in activity over a day will show a uniform activity across all intervals, which will be reflected in a large DDA_e and a small DDA_v. Similarly, by calculating across all animals, we could obtain the daily values of DDA_v and DDA_e for the group. This enabled us to examine the patterns and variations for animals across the experimental days.

### 2.4. Sensitivity Analysis

To determine the most suitable number of days for a reliable computation of the DDA measure, we conducted a sensitivity analysis. The sensitivity analysis proceeded by exploring overlapping periods of varying numbers of days from 3 to 6 in the Armidale dataset and from 3 to 19 in the Lansdown dataset. With an overlap of one day, there were five overlapping periods of three days, four of four, three of five, and two overlapping periods of six days in the Armidale dataset. Similarly, we calculated the DDA measure for overlapping periods in the Lansdown dataset. Due to the larger number of experiment days in the Lansdown dataset, the sensitivity analysis was expected to be more reliable compared to that on the Armidale dataset.

## 3. Results

### 3.1. Hourly Cattle Activity Profiles and Diurnal Patterns

**Figure 3:**
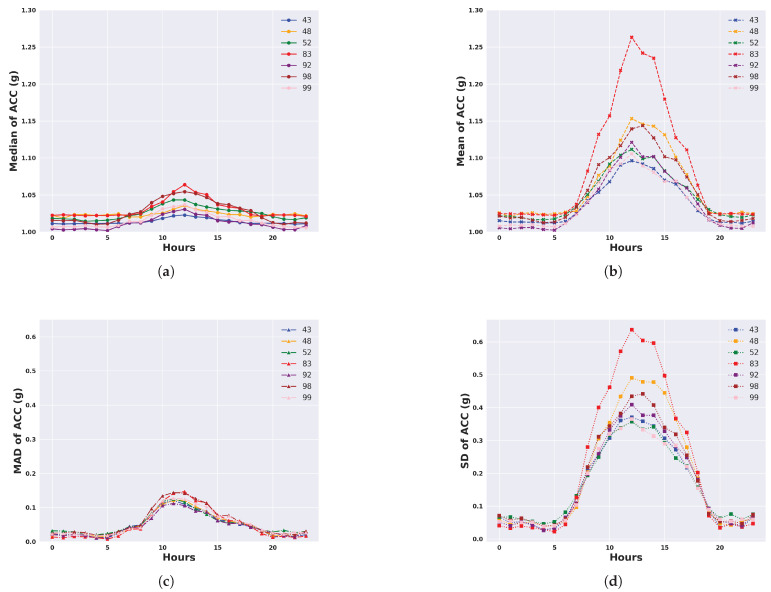
Representation of daily activity profiles when measured by different unfiltered features of accelerometer readings. Data are shown for each animal in the temperate environment of the Armidale trial and are averaged across all days. Each colour corresponds to an animal. (**a**) Activity measured by the median of the ACC; (**b**) Activity measured by the mean of the ACC; (**c**) Activity measured by the MAD of the ACC; (**d**) Activity measured by the SD of the ACC.

**Figure 4:**
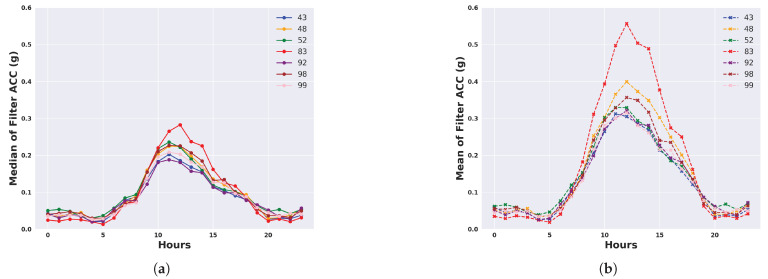

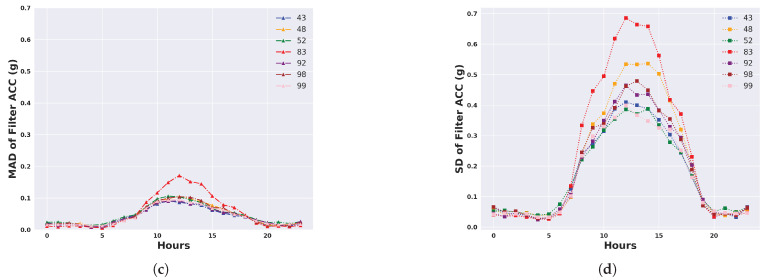
Representation of daily activity profiles when measured by different features following high-pass filtering to remove the effect of gravity from accelerometer readings. Data are shown for each animal in the temperate environment of the Armidale trial and are averaged across all days. Each colour corresponds to an animal. (**a**) Activity measured by the median of the filtered ACC; (**b**) Activity measured by the mean of the filtered ACC; (**c**) Activity measured by the MAD of the filtered ACC; (**d**) Activity measured by the SD of the filtered ACC.

**Figure 5:**
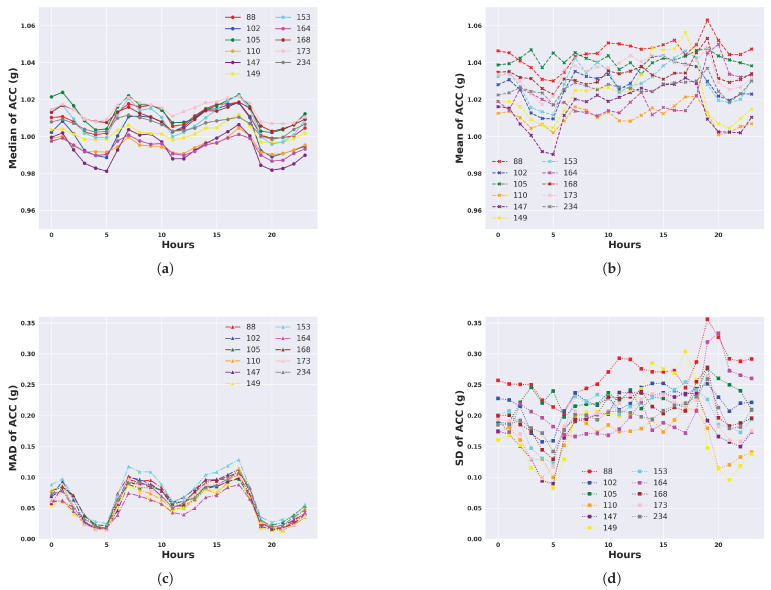
Representation of daily activity profiles when measured by different unfiltered features of accelerometer readings. Data are shown for each animal in the tropical environment of the Lansdown trial and are averaged across all days. Each colour corresponds to an animal. (**a**) Activity measured by the median of the ACC; (**b**) Activity measured by the mean of the ACC; (**c**) Activity measured by the MAD of the ACC; (**d**) Activity measured by the SD of the ACC.

**Figure 6:**
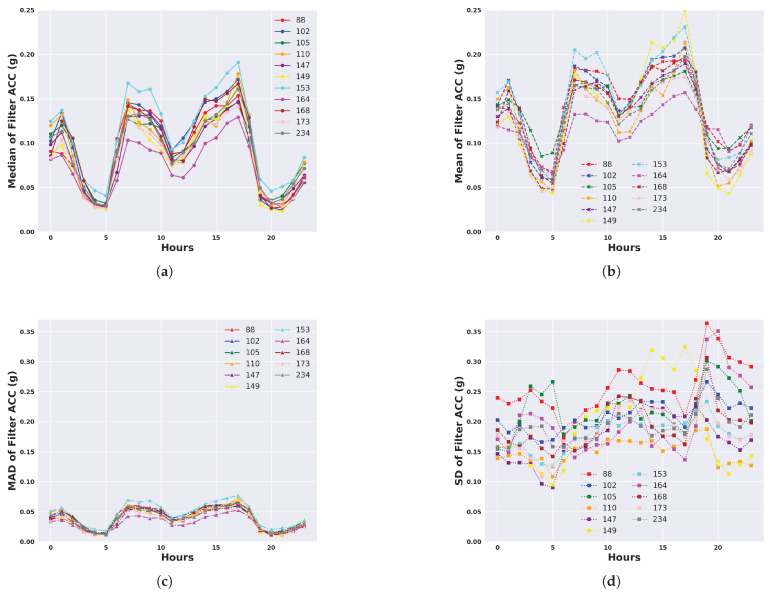
Representation of daily activity profiles when measured by different features calculated from high-pass-filtered accelerometer readings to remove the effect of gravity. Data are shown for each animal in the tropical environment of the Lansdown trial and are averaged across all days. Each colour corresponds to an animal. (**a**) Activity measured by the median of the filtered ACC; (**b**) Activity measured by the mean of the filtered ACC; (**c**) Activity measured by the MAD of the filtered ACC; (**d**) Activity measured by the SD of the filtered ACC.

We describe the 24 h activity profiles, calculated from both unfiltered and filtered acceleration data, for cattle in the temperate Armidale environment (Figure 3 and Figure 4) and tropical Lansdown environment (Figure 5 and Figure 6) experiments. Note the dominant effect of gravity observed in the median and mean of the unfiltered acceleration readings (Figure 3 and Figure 5) with a baseline of values near 1 g for each animal. However, when the median and mean were calculated from filtered data (Figures and Figure 6), the baseline values were near zero. This shows the importance of high-pass filtering to remove the effect of gravity from the accelerometer readings.

The activity profile of cattle in the temperate environment of the Armidale trial (Figure 3 and Figure 4) revealed activity at baseline levels between 00:00 to 06:00 and 20:00 to 23:59. Activity levels steadily increased from 06:00 until 12:00 and then declined at a slightly slower rate between 12:00 and 20:00. A similar activity profile was seen for each of the four calculated features although the magnitude (peaking at 0.5 to 0.7 g) was much higher for the standard deviation and the mean (Figure 4b,d).

The activity profile of cattle in the tropical environment of the Lansdown trial (Figure 5 and Figure 6) differed from that of the Armidale trial. In these data, three separate peaks of activity could be seen at 01:00, 07:00, and 17:00. The magnitude of the peaks was also lower than in Armidale (0.6–0.7 g as opposed to 0.1–0.2 g in Lansdown). Baseline levels of activity were observed at 03:00 to 05:00 and 19:00 to 21:00. A depression of activity was also observed between 11:00 to 13:00 but this did not fall to baseline levels, suggesting that a low level of activity was occurring. As shown in Figure 6, similar activity profiles were detectable for each of the four calculated features; however, the profile was most clear for the median based on the magnitude and consistency of the values between animals. Profiles derived from the SD or the mean were much more variable between animals. The results suggest that the median of the filtered acceleration yields consistent activity profiles between animals that may be indicative of diurnal activity patterns.

### 3.2. Cattle Daily Activity Profiles and the Trajectory of Activity

**Figure 7:**
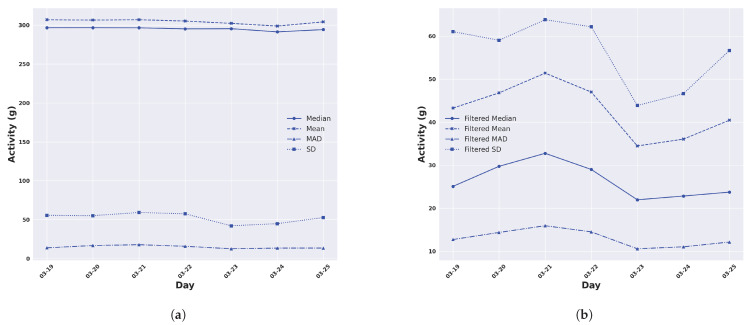
The daily activity in the temperate environment of Armidale averaged across all animals. (**a**) Activity measured by different features from the unfiltered ACC; (**b**) Activity measured by different features from the high-pass-filtered ACC.

**Figure 8:**
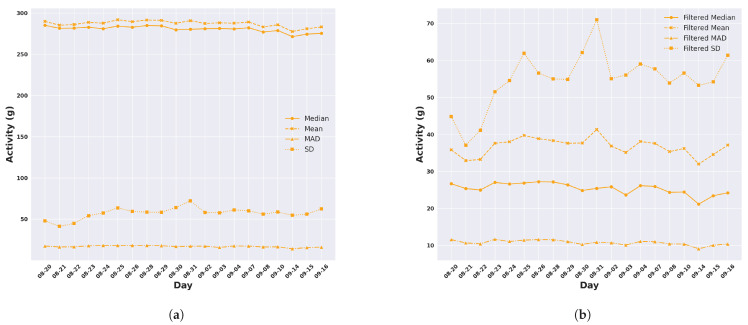
The daily activity in the tropical environment of Lansdown averaged across all animals. (**a**) Activity measured by different features from the ACC; (**b**) Activity measured by different features from the high-pass-filtered ACC.

In this section, we calculated the daily activity profile for each animal through the sum of the activity values for all five-minute windows within a day. The resulting values determined for each day were then averaged across all animals in the group and plotted for the duration of each trial (seven days in Armidale in Figure 7 and twenty days in Lansdown in Figure 8) to produce a trajectory of activity. This encompassed evaluating activity levels using the mean, median, SD, and MAD of unfiltered (Figure 7a and Figure 8b) and filtered (Figure 7b and Figure 8a) acceleration.

We observe that there are minimal fluctuations in the activity trajectory for each respective feature calculated from unfiltered accelerometer data for both Armidale and Lansdown trials. However, the variation in the activity trajectories can be seen in the features calculated from the filtered data (Figure 7b and Figure 8b). As an example (Figure 8b), the activity trajectory, as assessed by the standard deviation of the filtered acceleration in Lansdown, varied between 37.08 g and 70.99 g, whereas when measured by the median of filtered acceleration, it ranged from 21.14 g to 27.20 g. Similarly, in the Armidale trial, the daily activity values ranged from 43.85 g to 63.80 g for the SD and 21.95 g to 32.75 g for the median. At this stage, not enough data are available to explain why these changes in trajectory occur. Finally, the averaged total daily activity measured by the median of the filtered acceleration was surprisingly similar for cattle across both temperate and tropical environments, 26.43 g in the Armidale trial and 25.37 g in the Lansdown trial (Figure 7b and Figure 8b).

### 3.3. Daily Differential Activity

**Figure 9:**
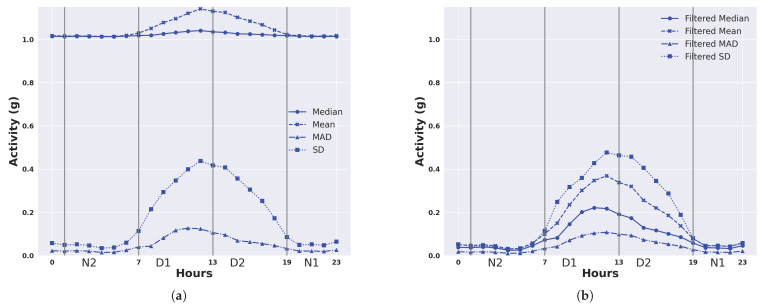
Average 24 h activity in Armidale (temperate). Vertical lines are the temporal boundaries for the case of four 6 h intervals: D1, D2, N1, N2. (**a**) Activity measured by different features of the ACC; (**b**) Activity measured by different features of the filtered ACC.

**Figure 10:**
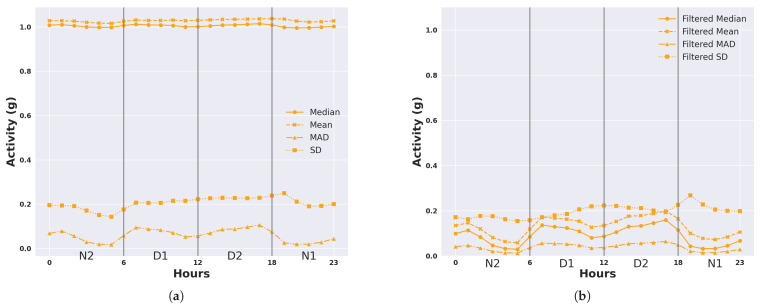
Average 24 h activity in Lansdown (tropical). Vertical lines are the temporal boundaries for the case of four 6 h intervals: D1, D2, N1, N2. (**a**) Activity measured by different features of the ACC; (**b**) Activity measured by different features of the filtered ACC.

Figure 9 and Figure 10 present the averaged 24 h activity in Armidale and Lansdown, illustrating overall trends across all animals. Vertical lines in these figures denote the boundaries for the four 6 h intervals: D1, D2, N1, and N2. These intervals serve as examples of how activity patterns vary throughout 24 h. We present the DDA results for four intervals as an example.

In Armidale (temperate), where animals were confined during the night and with little opportunity to graze, most of the activity was concentrated around midday. We present the activity measured by the unfiltered features and filtered features in Figure 9. The median and mean of unfiltered acceleration values were around 1 g. Based on the activity measured by the filtered features, most of the activity was concentrated in the periods D1 and D2. Conversely, in Lansdown (tropical), the animals had the freedom to move and graze at night, and there were three distinct peaks in activity levels at around midnight (01:00), early morning (07:00), and late afternoon (17:00) based on the median values of the filtered acceleration (see Figure 10b). We also noticed that the mean of the filtered acceleration had a similar trend to the median of the filtered acceleration in Lansdown.

**Figure 11:**
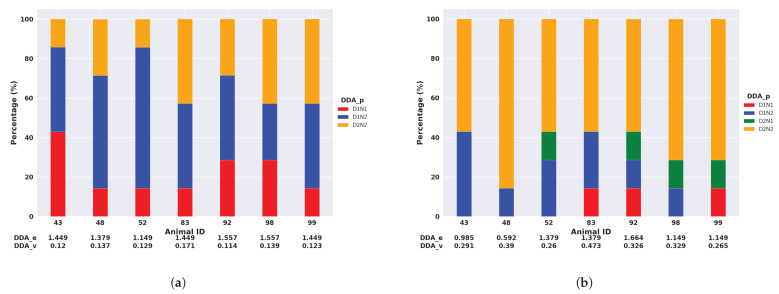
Bar plot of pairs for 4 intervals in Armidale. (**a**) Activity measured by the median of the filtered ACC; (**b**) Activity measured by the SD of the filtered ACC.

To assess the impact of different features on calculating DDA components, we utilised measurements from the median and the SD of the filtered acceleration. These features were employed in our case as they were the typical features demonstrating the activity in the previous results of circadian activity and daily activity. Figure 11 shows that only 3 out of 12 possible pairs were observed for the activity measured by the median of the filtered acceleration in Armidale. The animal with the ID of 52 had the least entropy (DDA_p), which means that this animal had the most regular activity levels (with a percentage of D1N2 over 70%). Even though animals 92 and 98 had the same entropy, we can see from DDA_v that animal 92 had a lower activity compared to animal 98.

**Figure 12:**
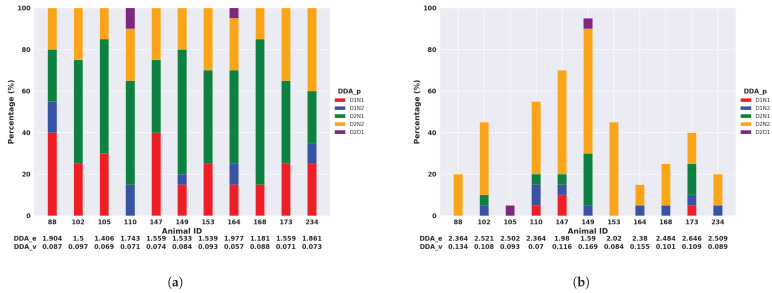
Bar plot of pairs for 4 intervals in Lansdown. (**a**) Activity measured by the median of the filtered ACC; (**b**) Activity measured by the SD of the filtered ACC.

The difference in DDA_p between the activity measured by the median and SD of the filtered acceleration in the Armidale data was not substantial. However, the Lansdown data showed significant variations between the two activity measurements. Figure 12 illustrates five pairs observed based on the activity measured by the median of the filtered acceleration, while a total of twelve pairs were observed based on the activity measured by the SD of the filtered acceleration.

Interestingly, three of the pairs observed in Armidale (D1N2, D2N2, and D1N1) also appeared in Lansdown. In Figure 12b, the same five pairs are observed based on the activity measured by the median of the filtered acceleration. In Figure 12b, it is also evident that the five most common pairs do not collectively account for 100% of the measured activity values. This is because there was much greater variation between animals in the most prominent DDA pair identified based on the standard deviation values. Additionally, we put the extra seven pairs observed based on the activity measured by the SD in Supplementary Figure S1.

Table 2 presents the percentage of DDA_p and DDA_v for both populations at four intervals (D1, D2, N1, and N2). The most frequently observed pair in Armidale was D1N2, accounting for 46.9% of the total occurrences according to activity measured by the median of the filtered acceleration. Conversely, in Lansdown, the predominant pair was D2N1, representing 45.5% of the total occurrences. These findings aligned with expectations as there was a significant increase in activity during the early morning (D1 to N2) in Armidale as the animals were yarded at night. However, in Lansdown, where the animals were free to roam and graze throughout the day and night, the most substantial activity shift occurred in the evening, from D2 to N1. The largest DDA_v values were all observed in D2N2 for both Armidale and Lansdown. The number of DDA_p values based on activity measured by the SD of the filtered acceleration were larger than those measured by the activity from the median of filtered acceleration. In the subsequent analysis, we employed the activity measured by the median of the filtered acceleration.

**Figure 13:**
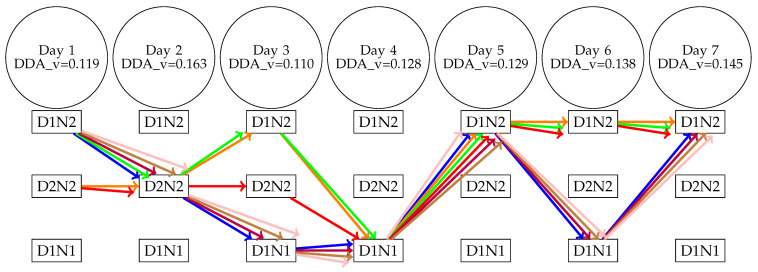
DDA_p for seven cattle from 4 intervals in Armidale, with different colours representing each cattle. The colours correspond to cattle IDs: blue for 43, orange for 48, green for 52, red for 83, purple for 92, brown for 98, and pink for 99.

Figure 13 illustrates the activity dynamics inferred by the DDA for the seven cattle in Armidale throughout the seven-day experiment when using four intervals to calculate the DDA values. Interestingly, on the first day of the experiment, D2N2 had the highest DDA_v value for only two animals (including animal 83). However, on the second day, it exhibited the highest value for all animals, but this continued into day 3 only for animal 83. D2N2 was not observed to have the highest value on any of the last four days. Equally remarkable, and possibly indicative of the social nature of cattle, is the observation that on four out of the seven days, only one pair had the highest DDA_v value. Moreover, animals 43 (blue), 92 (purple), 98 (brown), and 99 (pink) exhibited similar daily activity patterns. We present the DDA-induced activity dynamics of cattle for the Lansdown data in Supplementary Figures S2–S4. These figures show the daily activity changes for each animal.

### 3.4. Different Intervals

**Figure 14:**
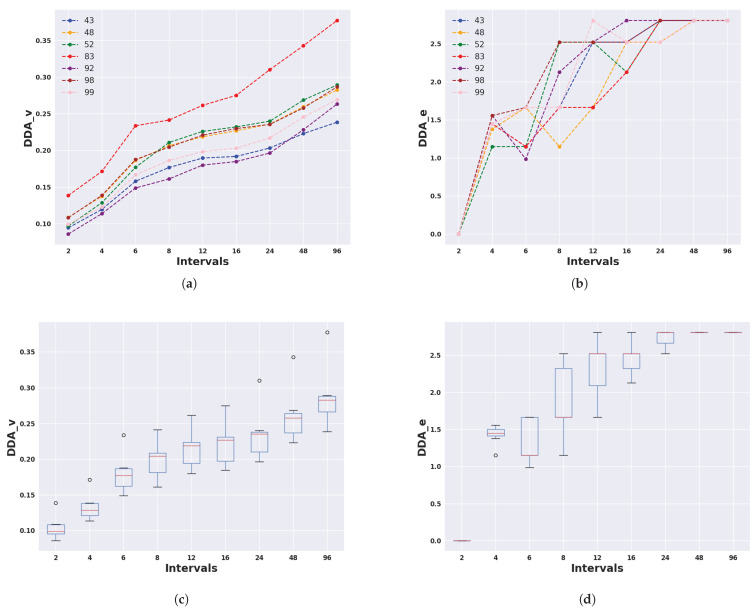
DDA_v (left) and DDA_e (right) values for each animal (top row) and across all animals (bottom row) for different numbers of daily intervals in the Armidale data. (**a**) DDA_v for each animal in different intervals; (**b**) DDA_e for each animal in different intervals; (**c**) DDA_v for all animals in different intervals; (**d**) DDA_e for all animals in different intervals.

**Figure 15:**
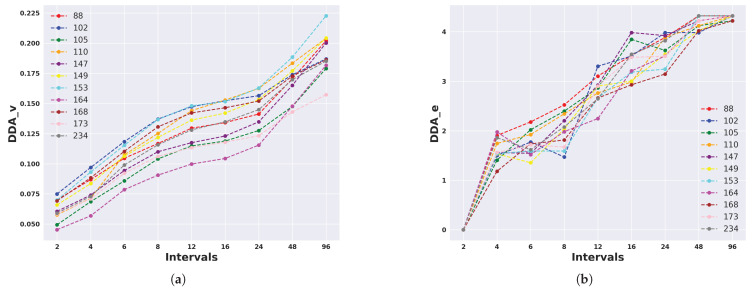

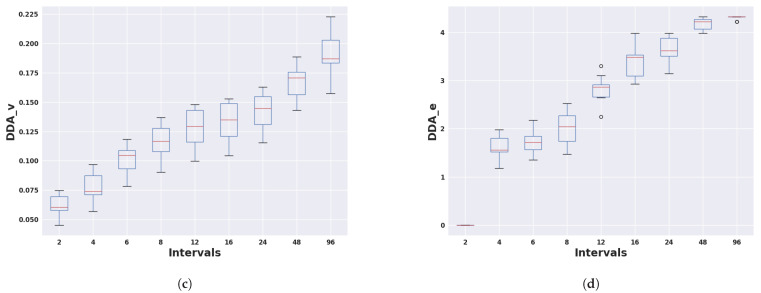
DDA_v (left) and DDA_e (right) values for each animal (top row) and across all animals (bottom row) for different numbers of daily intervals in the Lansdown data. (**a**) DDA_v for each animal in different intervals; (**b**) DDA_e for each animal in different intervals; (**c**) DDA_v for all animals in different intervals; (**d**) DDA_e for all animals in different intervals.

In order to find a more concise time period in which the highest and lowest activities occurred, we divided each 24 h considering nine different interval numbers/sizes. We present DDA_v and DDA_e values for Armidale and Lansdown data considering 2, 4, 6, 8, 12, 16, 24, 48, and 96 intervals in Figure 14 and Figure 15. DDA_v values for Armidale data were notably higher than those for Lansdown data. This discrepancy could mainly be attributed to differences in management practices, specifically whether the animals were penned overnight. This disparity likely resulted in an artificial inflation of the activity levels between day and night.

For both Armidale and Lansdown data, DDA_v demonstrated an increase as the number of intervals increased. This is because having more intervals with shorter duration enhanced the sensitivity to identify the precise times when the animals exhibited their highest levels of activity (see Figure 14 and Figure 15). As the number of intervals increased, DDA_e reached a plateau at a value that was equal to the logarithm base two of the number of the experiment days, which corresponded to scenarios where animals exhibited entirely different DDA_p patterns on each of the experiment days. This translated to values of $\log_{2}7\approx 2.807$ and $\log_{2}20\approx 4.322$ for Armidale and Lansdown data, respectively. This observation highlights a trade-off between DDA_v and DDA_e, suggesting that an optimal number of intervals and a minimum number of experiment days may be required to obtain robust and informative values. To further ascertain the relationship between the number of days and the DDA measures, in the next subsection, we conduct a further analysis by considering windows of consecutive days with various lengths.

### 3.5. Sensitivity Analysis

**Figure 16:**
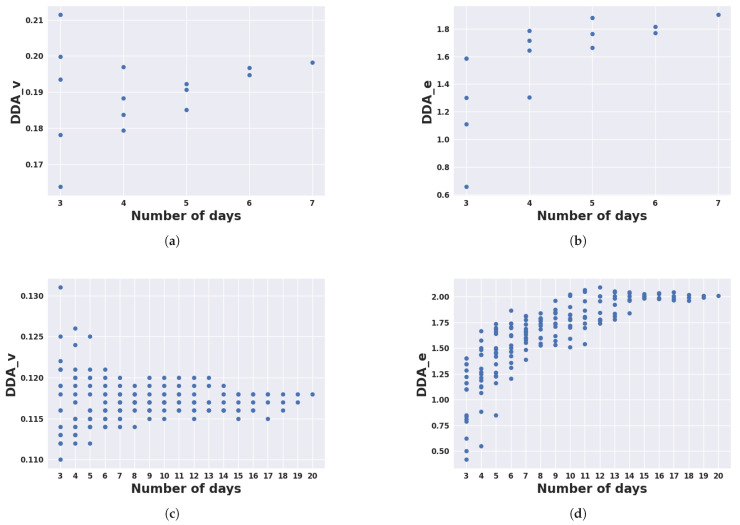
Sensitivity analysis of DDA_v (left) and DDA_e (right) values for Armidale (top row) and Lansdown (bottom row) data by increasing the number of days whose data were used for calculating the DDA measures. (**a**) DDA_v values for different numbers of days in Armidale data; (**b**) DDA_e values for different numbers of days in Armidale data; (**c**) DDA_v values for different numbers of days in Lansdown data; (**d**) DDA_e values for different numbers of days in Lansdown data.

Figure 16 illustrates the results of our sensitivity analysis exploring windows of consecutive days with lengths ranging from 3 days to 6 days in the Armidale data and 3 days to 19 days in the Lansdown data. The results for the Armidale data were inconclusive due to the limited number of total days (i.e., 7 days). In the sensitivity analysis, the initial days of the experiment were over-represented in the consecutive intervals. Therefore, anomalies in the initial days may have introduced bias in the results. In the Lansdown dataset, which contained a higher number of days and animals, the sensitivity analysis results showed a more consistent pattern of convergence toward the final value observed when all days were considered.

## 4. Discussion

Understanding animal activity levels or trajectories may be useful for monitoring animal health, welfare, and productivity, but these applications require validated and reliable approaches to assess activity. In this study, we assessed the utility of different statistical features and data preprocessing methods to calculate cattle activity from triaxial accelerometer readings. The resulting data were reported on an hourly or daily basis to provide insights into diurnal or temporal activity patterns.

Triaxial accelerometer readings were transformed into measures of animal activity by the calculation of different statistical features. A common approach is to compute the magnitude of the acceleration from three-dimensional accelerometer data, and the associated statistics, such as the mean and standard deviation, which can serve as a proxy for overall activity intensity and to characterise activity patterns. The choice of the statistical feature depends on the desired level of robustness to handle outliers or irregularities in the data [29] but must also ensure accurate diagnostic capacity. To investigate activity trends over time, researchers often segment the data into specific window sizes and extract features from each window size [33]. Sarout et al. [3] used the motion index provided by an Icetag Pro logger to measure sheep circadian rhythm of activity, which they defined as the average of the magnitude of the acceleration on each of the three axes for each minute. There are few papers that examine the impact of features from accelerometer data on the calculation of activity, which highlights the importance of the current study.

The method chosen for measuring animal activity, whether it involves filtered values or specific statistics (e.g., mean, standard deviation, median, or MAD), can significantly influence the resulting 24 h activity. A high-pass filter is a preprocessing step for the accelerometer data analysis that removes the influence of gravity. We evaluated the effects of the four statistical features of the filtered acceleration to measure activity. We utilised the median and SD of the filtered acceleration to calculate the DDA components because the activity measured by these two features exhibited distinct patterns for the 24 h activity. Similarly, the results of DDA_p and the number of pairs were different based on the different activity measurements, especially in the Lansdown dataset. The consistent appearance of certain pairs (D1N2, D2N2, and D1N1) in the analysis results of both datasets indicates that specific behavioural patterns may be inherent to particular animals, regardless of the location or measurement method. These insights emphasise the importance of a careful selection of the activity measurement method to ensure an accurate activity level interpretation and comparison of DDA_p results across different datasets and animal populations. Therefore, a careful consideration of filtering processing techniques is needed for the activity measured from accelerometer data.

Our study facilitates the identification of diurnal rhythms and periodic fluctuations in activity levels. Multiple factors, encompassing breed, management practices, and environmental conditions, contribute to variations in animal behaviour and activity levels as observed through the examination of activity patterns. For example, in another study, cattle subjected to different management practices exhibited noticeable variations in their circadian activity patterns encompassing variances in peak activity times and overall activity levels. These differences could be attributed to breed-specific characterisations, such as grazing/foraging behaviour, metabolic rates, or genetic predispositions [42].

Generally, distinct patterns emerged for Armidale and Lansdown, regardless of the feature used in the activity calculation. For example, cattle kept in the tropical climate of Lansdown displayed three daily peaks of activity occurring at 01:00, 07:00, and 17:00, whereas cattle kept in the temperate Armidale climate showed a single peak at 12:00. Further, the intensity of motion calculated for these peaks was different, with peaks in the tropical climate recorded at 0.2 g and that of the temperate climate at 0.7 g. The disparity in activity patterns between the two locations can be attributed to the cattle being confined in pens during the night in Armidale. In contrast, they were free to graze at all times in Lansdown. This difference in management practices had the potential to significantly impact the activity levels observed. Furthermore, the median of the filtered acceleration appeared to be a more consistent feature for measuring activity compared to the SD of the filtered acceleration, particularly for the Lansdown data. This is because activity profiles generated from the standard deviation of the filtered acceleration exhibited limited consistency among animals and greater overall variation in values. However, it is important to consider that our goal in this study was to define the consistent aspects of activity or diurnal activity profiles. In other studies focused on the detection of disease or injury, it is possible that a feature such as the SD may be more informative as it is rapidly responsive to changes in the distribution of accelerometer force readings. Our analysis of the results suggests that management practices can influence activity patterns.

In addition to breed and management practices, environmental and climate factors also have an important impact on variations in activity. Variables such as temperature, daylight duration, and seasonal shift likely influence animal behaviour, contributing to variances in their activity patterns [3]. Further research in this domain is essential for a comprehensive understanding of how these variables intricately shape animal diurnal activity patterns.

## 5. Conclusions

We showed that different measures of animal activity including hourly, daily, or differential activity levels within a 24 h period could be calculated from triaxial accelerometer readings. By preprocessing the data through high-pass filtering and subsequently calculating the Euclidean norm, we effectively minimised the impact of gravity and improved the ability to discern activity patterns within the data. We found that the median was a reliable feature for observing diurnal activity patterns that are consistently exhibited by different animals within a specific environment. Differences in the diurnal patterns were evident among cattle from the two different field sites. Further research is necessary to delve into the underlying factors contributing to this disparity. Sensors such as accelerometers embedded in ear tags have great potential for automating the monitoring of animal health, welfare, and productivity in the animal production sector. However, the successful application of these approaches requires a deep understanding of typical activity profiles to ensure the accurate detection of anomalies.

## Figures and Tables

**Table 1:** The number of pairs for 2, 4, 6, 8, 12, 16, 24, 48, and 96 intervals within 24 h.

Intervals	Exemplification	#Pairs
2	D, N	2
4	D1, D2, N1, N2	12
6	D1, …, D3, N1, …, N3	30
8	D1, …, D4, N1, …, N4	56
12	D1, …, D6, N1, …, N6	132
16	D1, …, D8, N1, …, N8	240
24	D1, …, D12, N1, …, N12	552
48	D1, …, D24, N1, …, N24	2256
96	D1, …, D48, N1, …, N48	9120

**Table 2:** The percentage of DDA_p and DDA_v of 4 intervals for Armidale (temperate) and Lansdown (tropical). Filtered median denotes the activity measured by the median of the filtered ACC. Filtered SD denotes the activity measured by the SD of the filtered ACC.

	Armidale				Lansdown			
	**Filtered Median**		**Filtered SD**		**Filtered Median**		**Filtered SD**	
	**Percent (%)**	**DDA_v (g)**	**Percent (%)**	**DDA_v (g)**	**Percent (%)**	**DDA_v (g)**	**Percent (%)**	**DDA_v (g)**
D1D2	NA	NA	NA	NA	NA	NA	1.8	0.062
D1N1	22.4	0.124	6.1	0.323	23.2	0.072	1.8	0.070
D1N2	46.9	0.131	20.4	0.333	5.0	0.074	4.1	0.064
D2D1	NA	NA	NA	NA	1.4	0.052	0.9	0.074
D2N1	NA	NA	8.2	0.319	45.5	0.081	5	0.130
D2N2	30.6	0.142	65.3	0.336	25	0.082	27.7	0.126
N1D1	NA	NA	NA	NA	NA	NA	11.8	0.115
N1D2	NA	NA	NA	NA	NA	NA	11.4	0.104
N1N2	NA	NA	NA	NA	NA	NA	25.5	0.118
N2D1	NA	NA	NA	NA	NA	NA	3.6	0.103
N2D2	NA	NA	NA	NA	NA	NA	4.5	0.085
N2N1	NA	NA	NA	NA	NA	NA	1.8	0.075

## Data Availability

Data can be made available upon request.

## Supplementary Materials

The following supporting information can be downloaded at: www.mdpi.com/xxx/s1, Table S1: Temperature statistics during the experiments at Armidale and Lansdown; Figure S1: Barplot of pairs for 4 intervals in Lansdown; Figure S2: The DDA_p for seven cattle daily in Lansdown, with different colours representing each cattle. The colours correspond to Cattle IDs: red for 88, blue for 102, green for 105, orange for 110, purple for 147, yellow for 149, cyan for 153, magenta for 164, brown for 168, pink for 173 and grey for 234; Figure S3: The DDA_p for seven cattle daily in Lansdown, with different colours representing each cattle. The colours correspond to Cattle IDs: red for 88, blue for 102, green for 105, orange for 110, purple for 147, yellow for 149, cyan for 153, magenta for 164, brown for 168, pink for 173 and grey for 234; Figure S4: The DDA_p for seven cattle daily in Lansdown, with different colours representing each cattle. The colours correspond to Cattle IDs: red for 88, blue for 102, green for 105, orange for 110, purple for 147, yellow for 149, cyan for 153, magenta for 164, brown for 168, pink for 173 and grey for 234.
