# Supplementary material for: Analyzing Cattle Activity Patterns with Ear Tag Accelerometer Data
Source: Animals (Basel). 2024 Jan 18;14(2):0. doi: 10.3390/ani14020301 (PMC11154254; doi:10.3390/ani14020301)
Supplement: Supplementary file 1 [file animals-14-00301-s001.zip › animals-2712809-supplementary.pdf]

Supplementary Materials: Analyzing Cattle Activity Patterns with Ear Tag Accelerometer Data

Shuwen Hu, Antonio Reverter, Reza Arablouei, Greg Bishop-Hurley, Jody McNally, Flavio Alvarenga, and Aaron Ingham

Table S1. Temperature statistics during the experiments at Armidale and Lansdown.

	March 2020	August/September 2020
average minimum temperature	4.5 °C	10.3 °C
average maximum temperature	27.4 °C	32.3 °C
average temperature	16.0 °C	21.9 °C

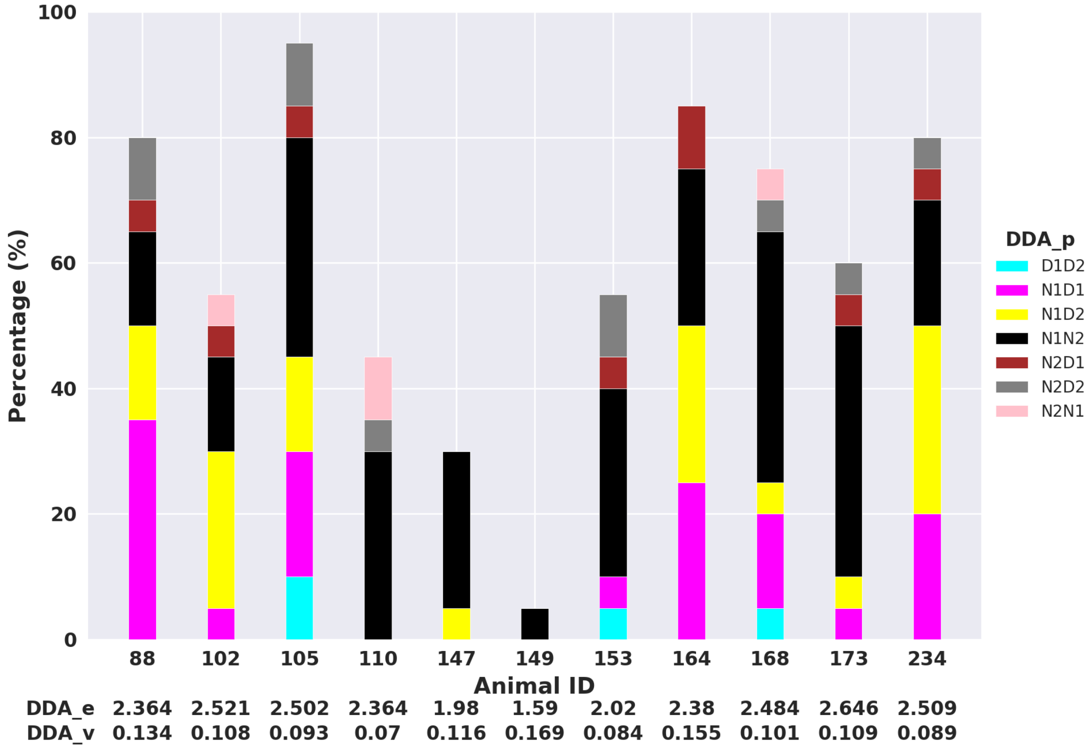

Figure S1. Barplot of pairs for 4 intervals in Lansdown.

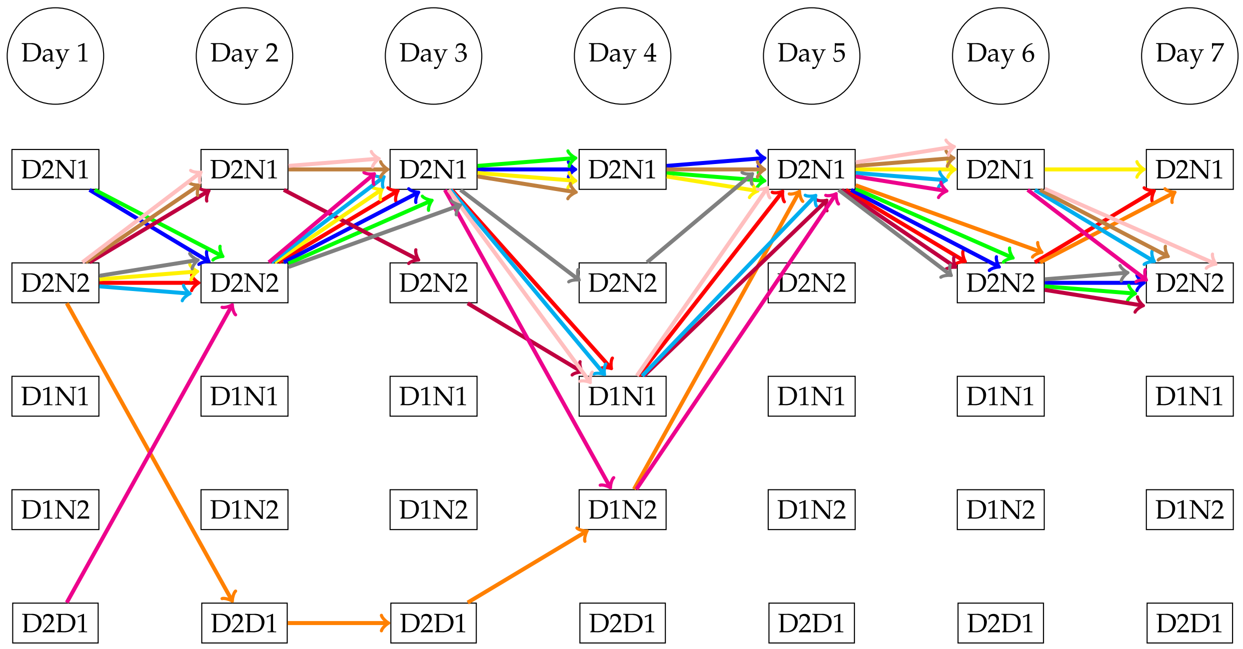

Figure S2. The DDA_p for seven cattle daily in Lansdown, with different colours representing each cattle. The colours correspond to Cattle IDs: red for 88, blue for 102, green for 105, orange for 110, purple for 147, yellow for 149, cyan for 153, magenta for 164, brown for 168, pink for 173 and grey for 234.

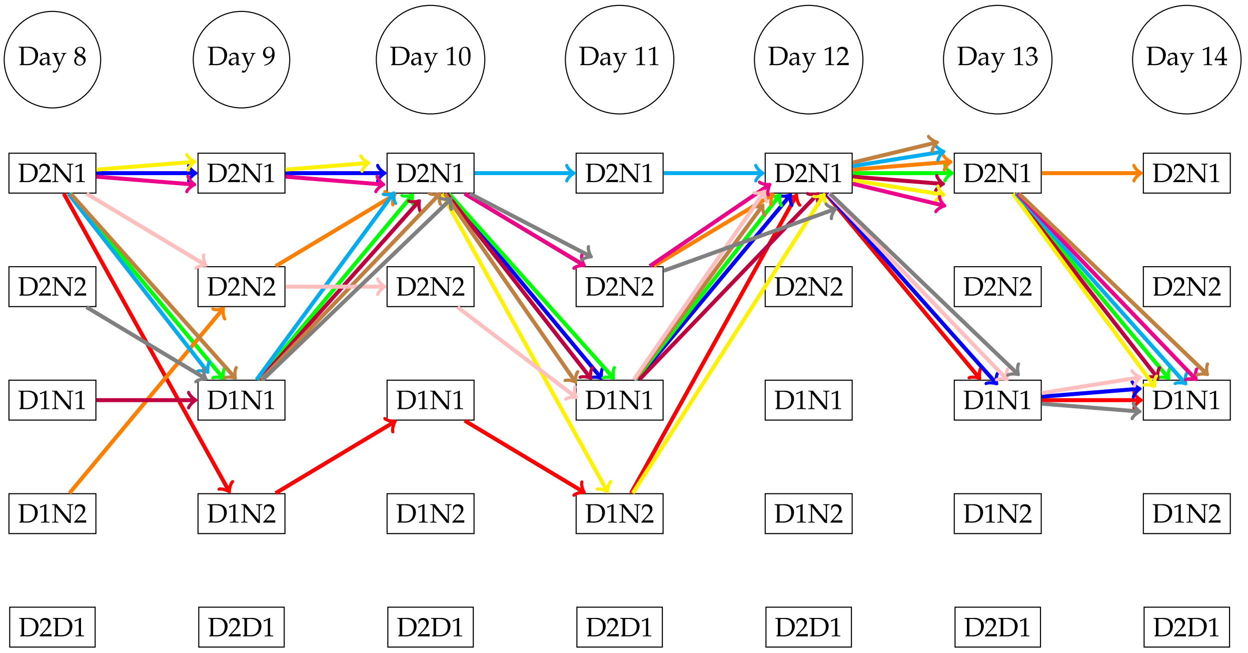

Figure S3. The DDA_p for seven cattle daily in Lansdown, with different colours representing each cattle. The colours correspond to Cattle IDs: red for 88, blue for 102, green for 105, orange for 110, purple for 147, yellow for 149, cyan for 153, magenta for 164, brown for 168, pink for 173 and grey for 234.

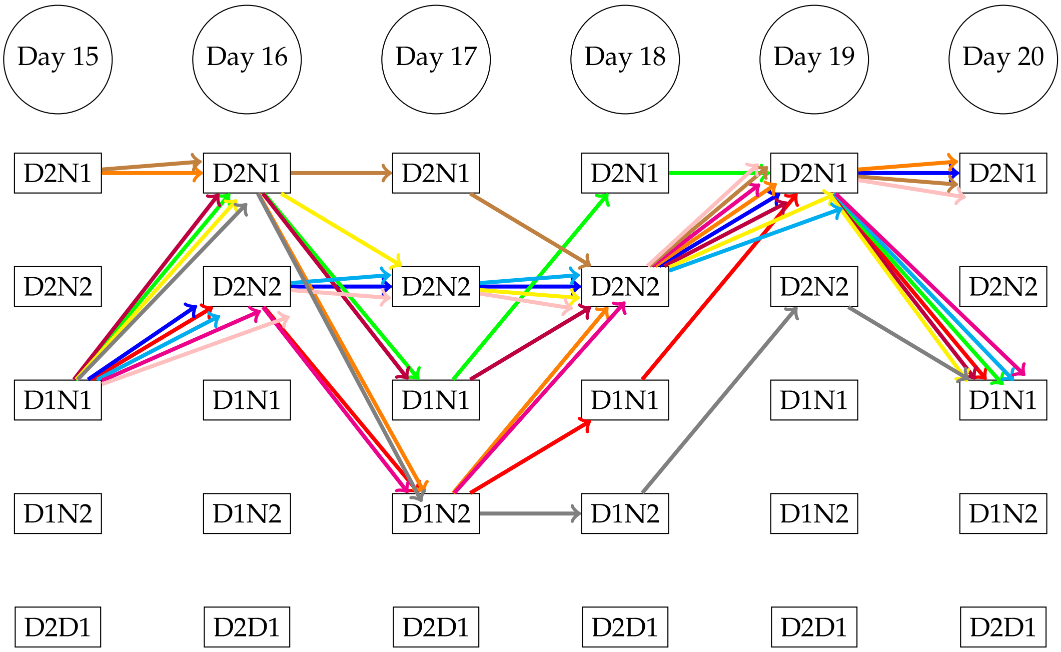

Figure S4. The DDA_p for seven cattle daily in Lansdown, with different colours representing each cattle. The colours correspond to Cattle IDs: red for 88, blue for 102, green for 105, orange for 110, purple for 147, yellow for 149, cyan for 153, magenta for 164, brown for 168, pink for 173 and grey for 234.
